# Supplementary figures and images for: A novel bivalent interaction mode underlies a non-catalytic mechanism for Pin1-mediated protein kinase C regulation
Source: eLife. 2024 Apr 30;13:e92884. doi: 10.7554/eLife.92884 (PMC11060717; doi:10.7554/eLife.92884)

Figure 7A actin source data

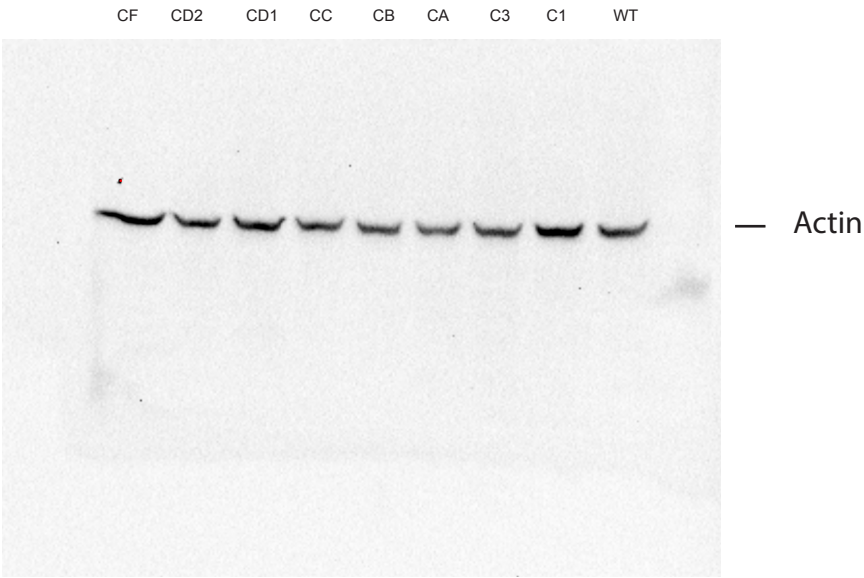

Supplement: Figure 7—source data 1. [file elife-92884-fig7-data1.zip › Figure 7-source data1 /F7A actin/F7A actin source data labeled.pdf]

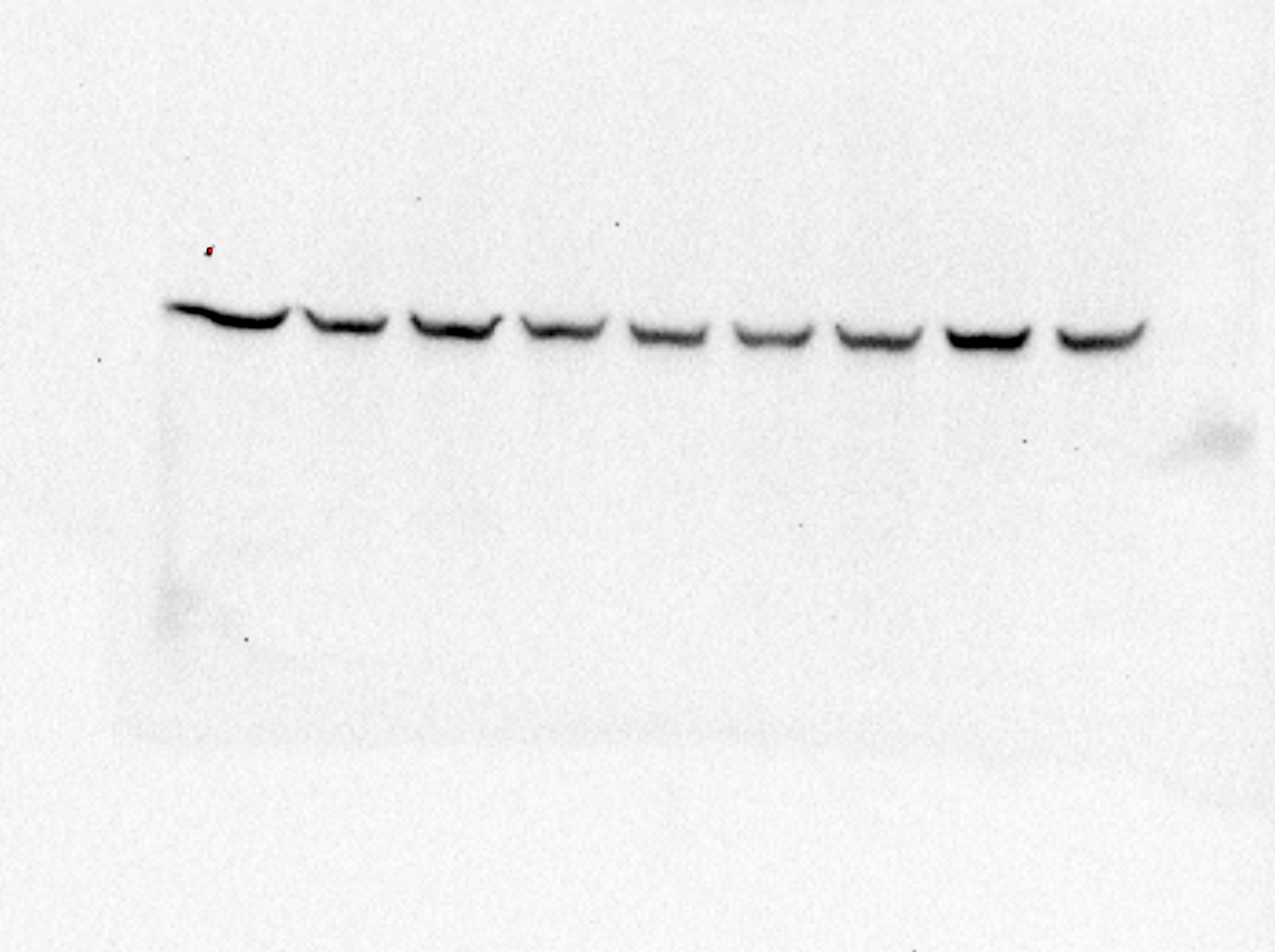

Supplement: Figure 7—source data 1. [file elife-92884-fig7-data1.zip › Figure 7-source data1 /F7A actin/F7A actin source data unlabeled.tif]

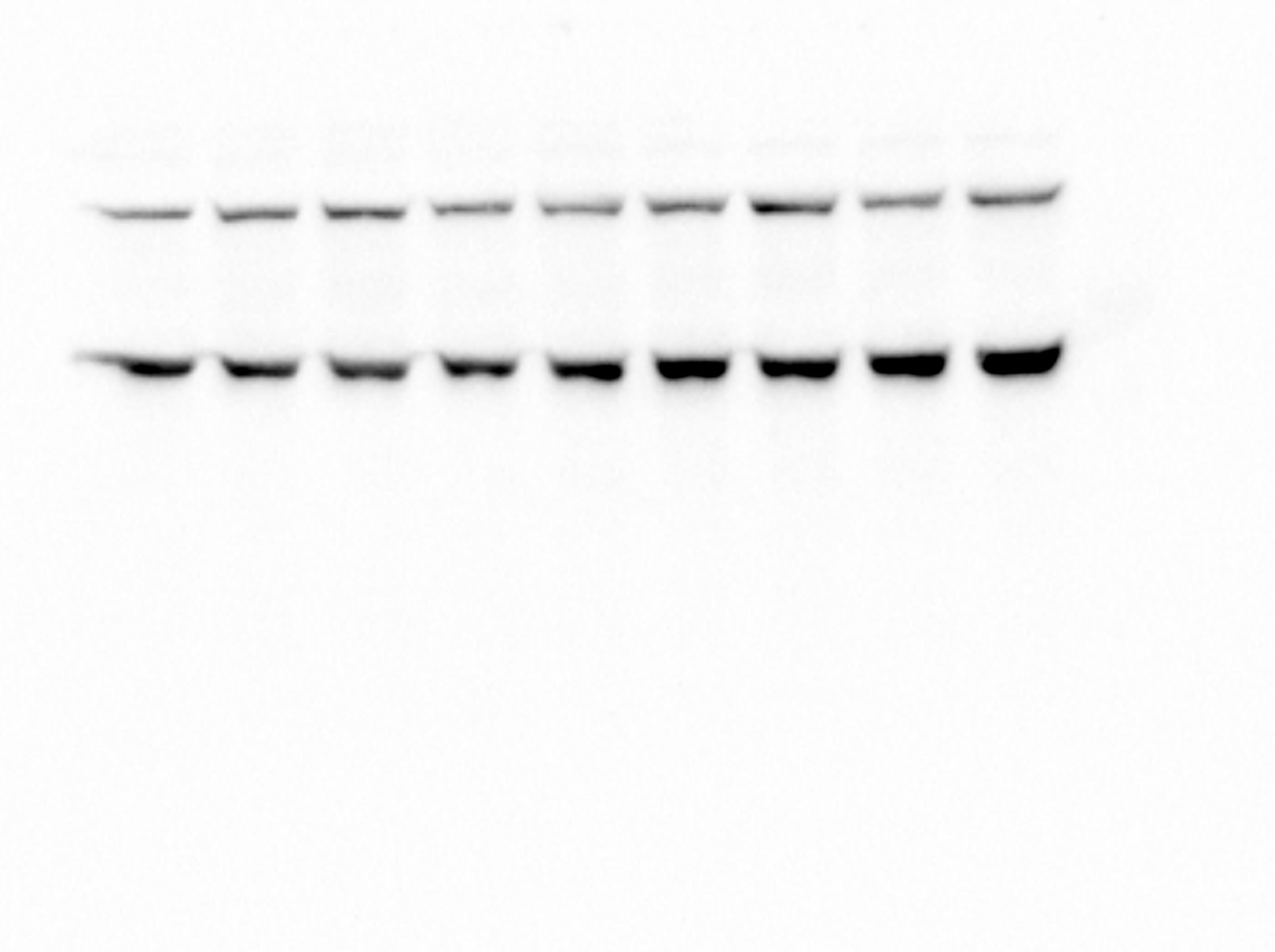

Supplement: Figure 7—source data 1. [file elife-92884-fig7-data1.zip › Figure 7-source data1 /F7A PKC alpha/F7A PKC alpha source data unlabeled 2.tif]

CF CD2 CD1 CC CB CA C3 C1 WT

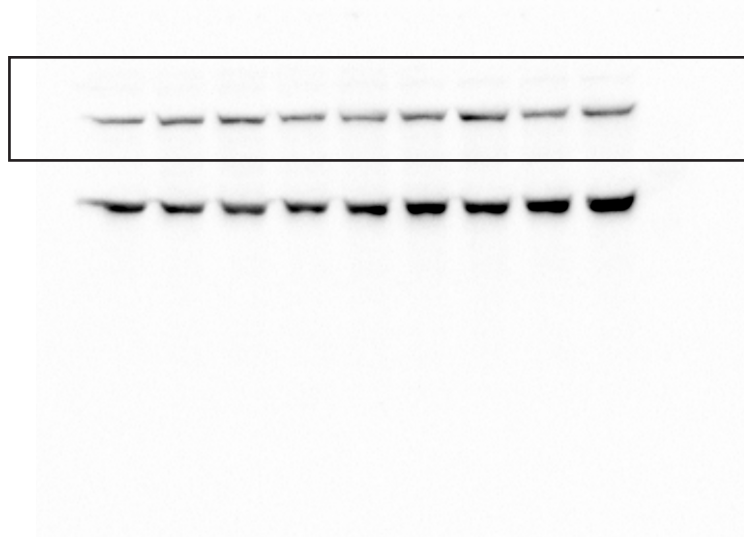

PKC alpha

Supplement: Figure 7—source data 1. [file elife-92884-fig7-data1.zip › Figure 7-source data1 /F7A PKC alpha/F7A PKC alpha source data labeled 2.pdf]

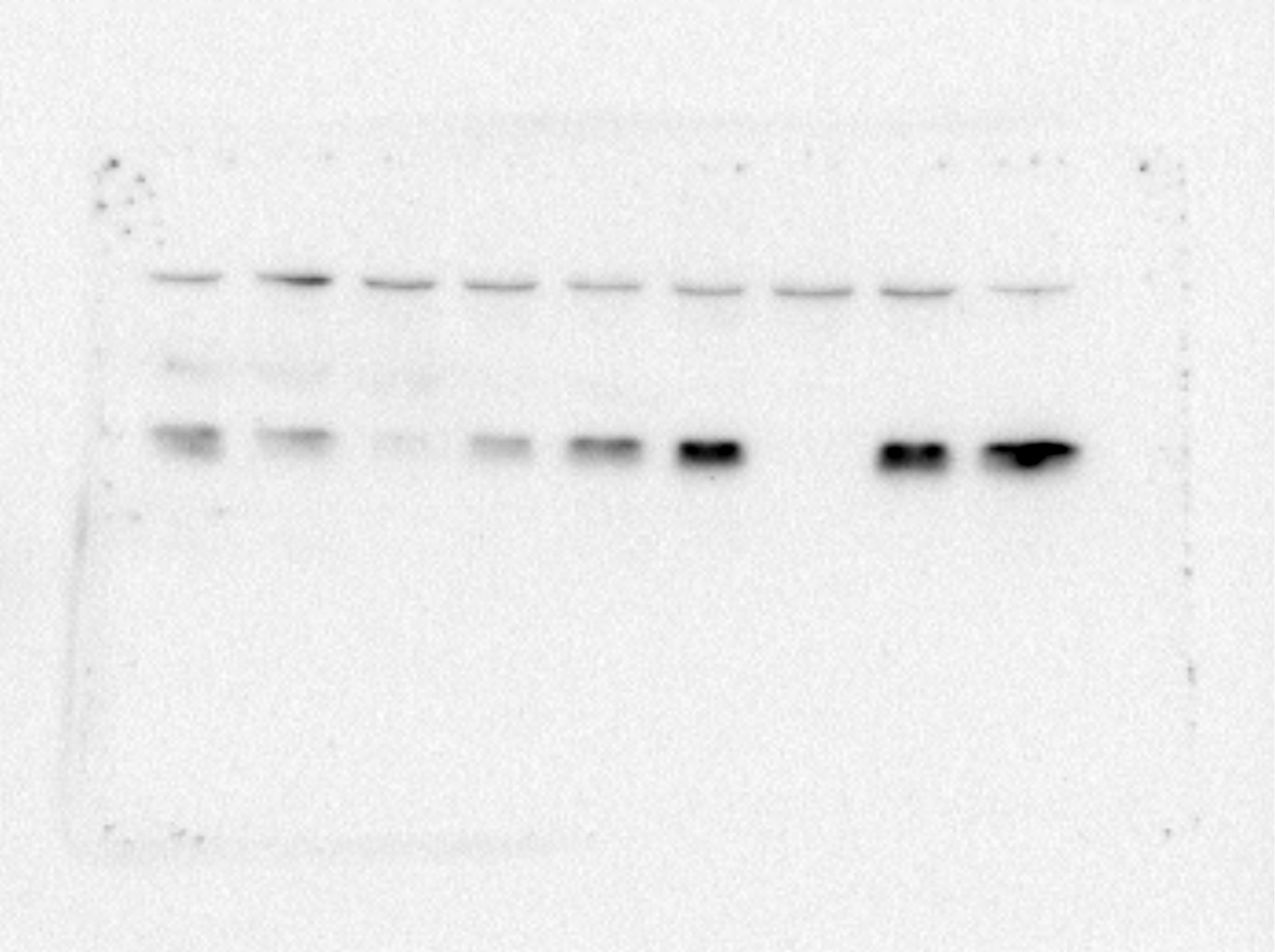

Supplement: Figure 7—source data 1. [file elife-92884-fig7-data1.zip › Figure 7-source data1 /F7A Pin1/F7A Pin1 source data unlabeled.tif]

Figure 7A Pin1 source data

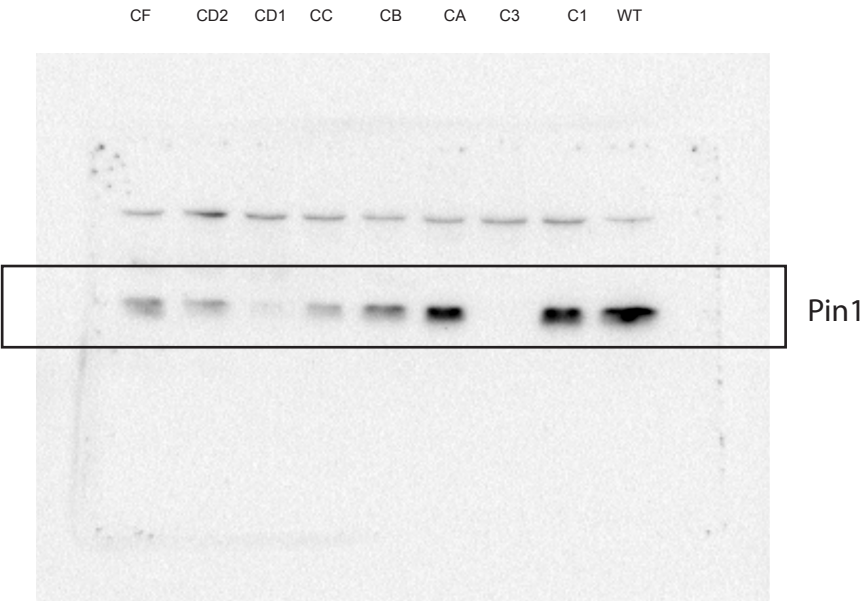

Supplement: Figure 7—source data 1. [file elife-92884-fig7-data1.zip › Figure 7-source data1 /F7A Pin1/F7A Pin1 source data labeled.pdf]

Figure 7C lower panel Pin1 source data

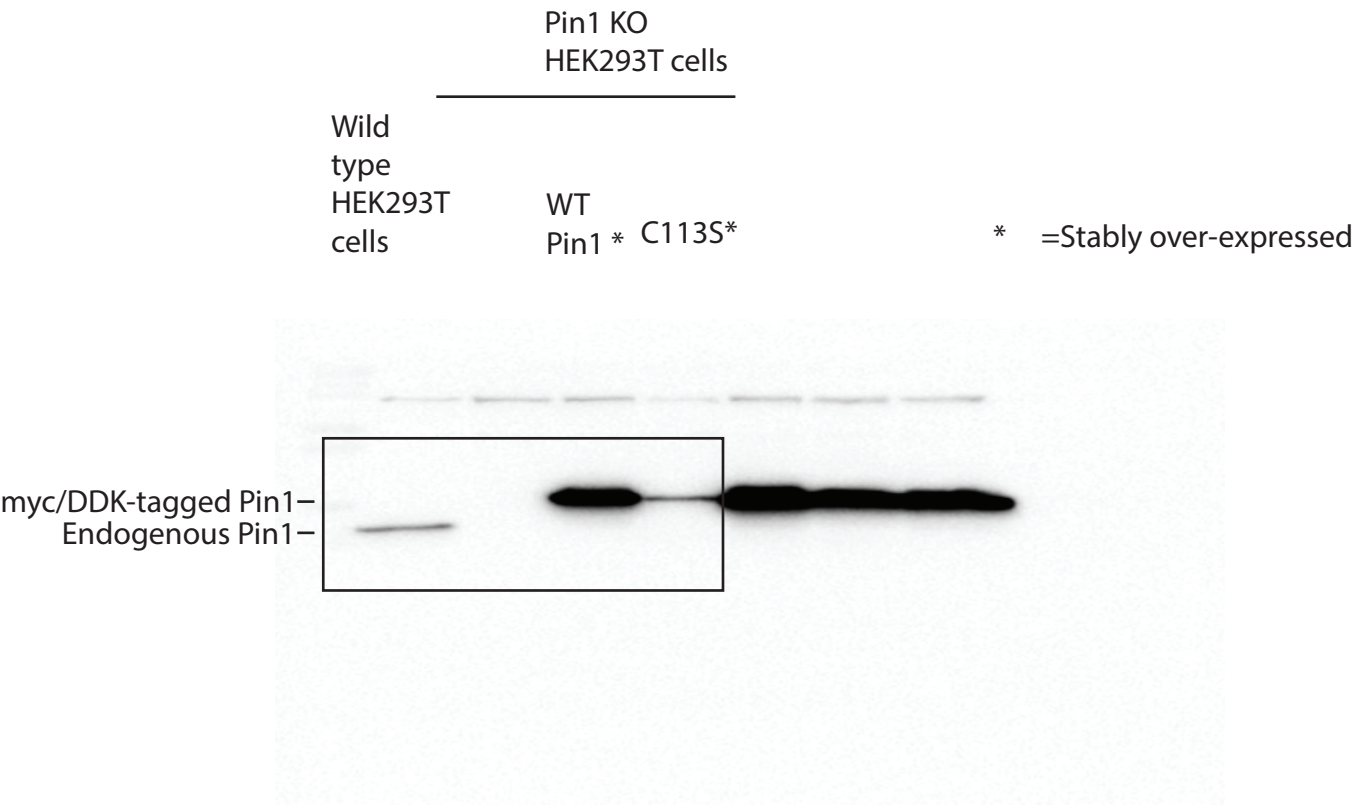

Supplement: Figure 7—source data 2. [file elife-92884-fig7-data2.zip › Figure 7-source data2/F7C Lower Panel/Pin1/F7C lower panel source data Pin1 labeled .pdf]

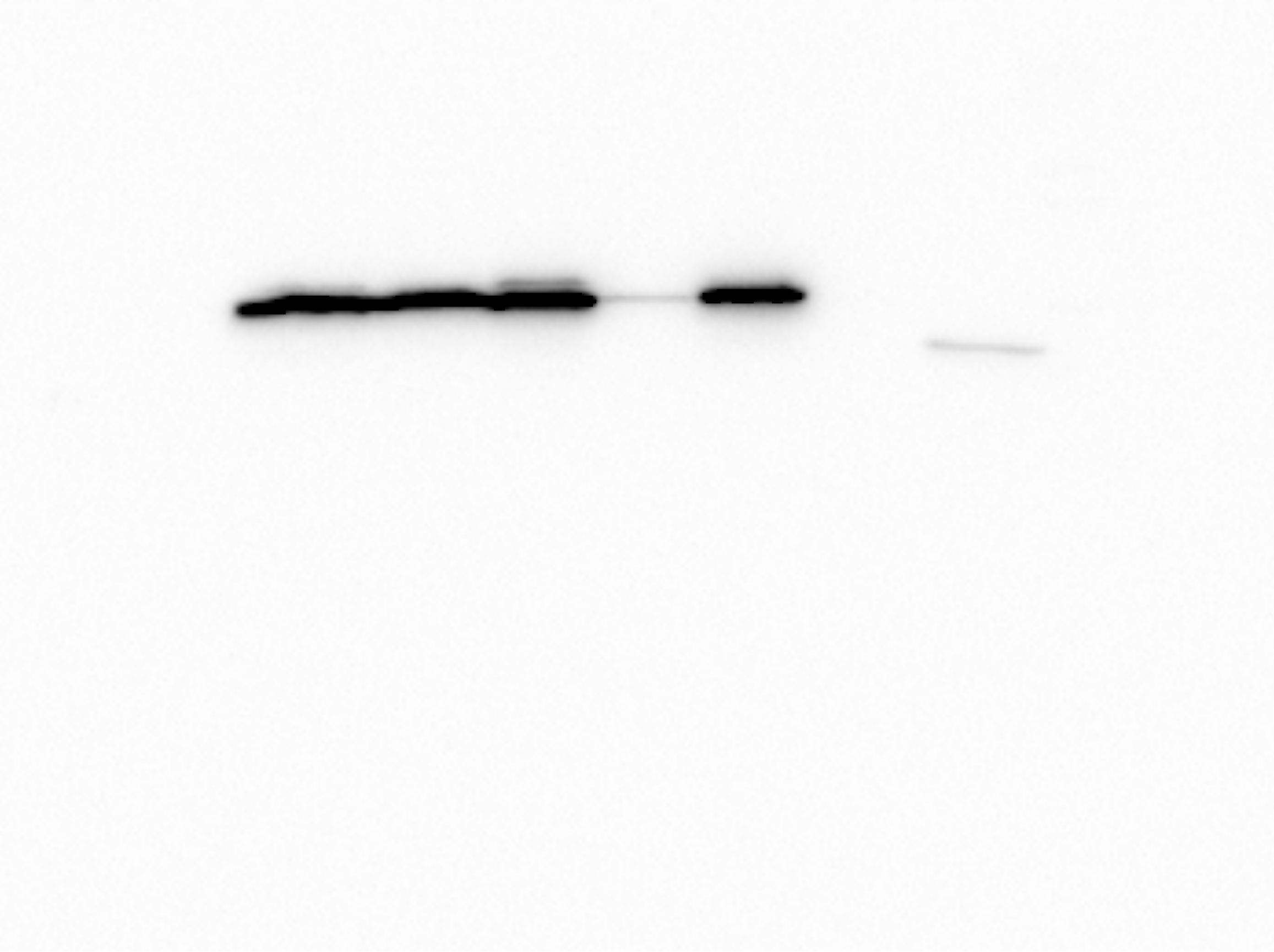

Supplement: Figure 7—source data 2. [file elife-92884-fig7-data2.zip › Figure 7-source data2/F7C Lower Panel/Pin1/F7C lower panel Pin1 unlabeled.tif]

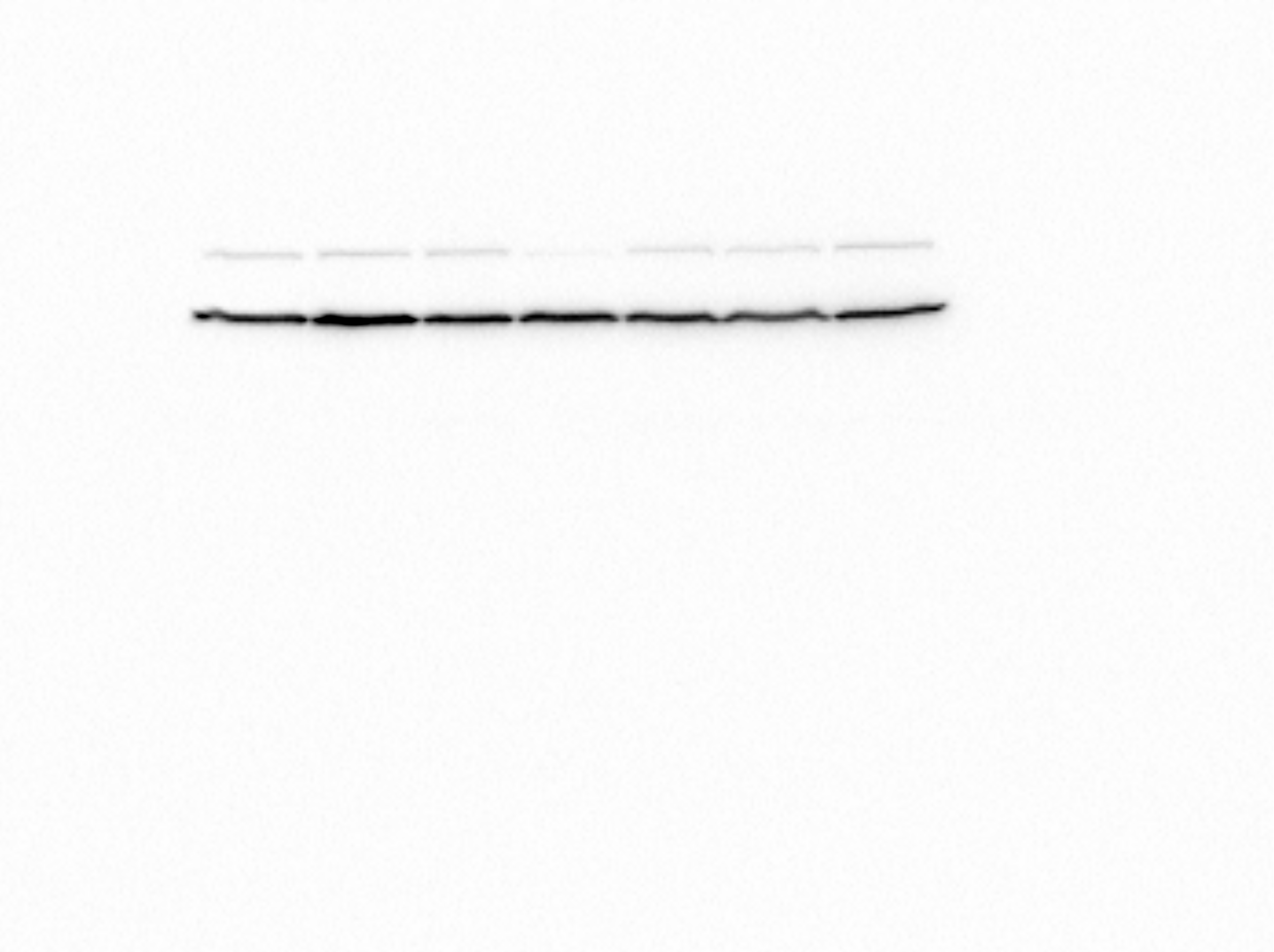

Supplement: Figure 7—source data 2. [file elife-92884-fig7-data2.zip › Figure 7-source data2/F7C Lower Panel/Actin/F7C lower panel unlabeled actin.tif]

Figure 7C lower panel actin source data

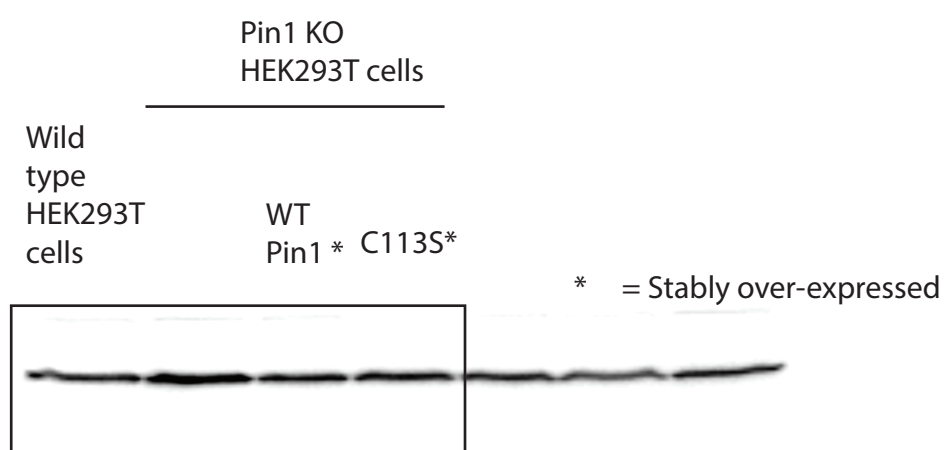

Supplement: Figure 7—source data 2. [file elife-92884-fig7-data2.zip › Figure 7-source data2/F7C Lower Panel/Actin/F7C lower panel source data actin labeled .pdf]

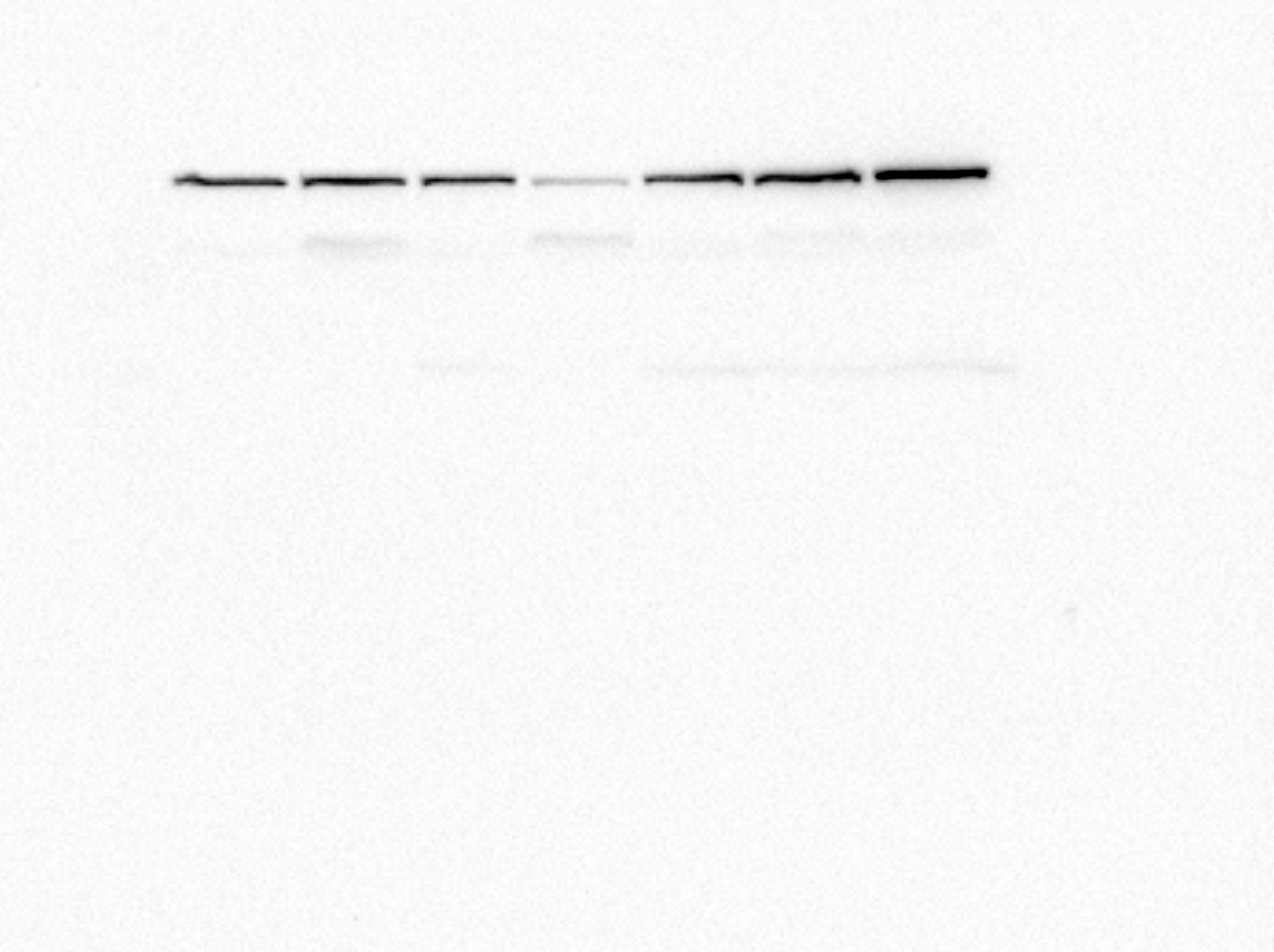

Supplement: Figure 7—source data 2. [file elife-92884-fig7-data2.zip › Figure 7-source data2/F7C Lower Panel/PKC alpha/F7C lower panel PKC alpha unlabeled.tif]

Figure 7C lower panel PKC alpha source data

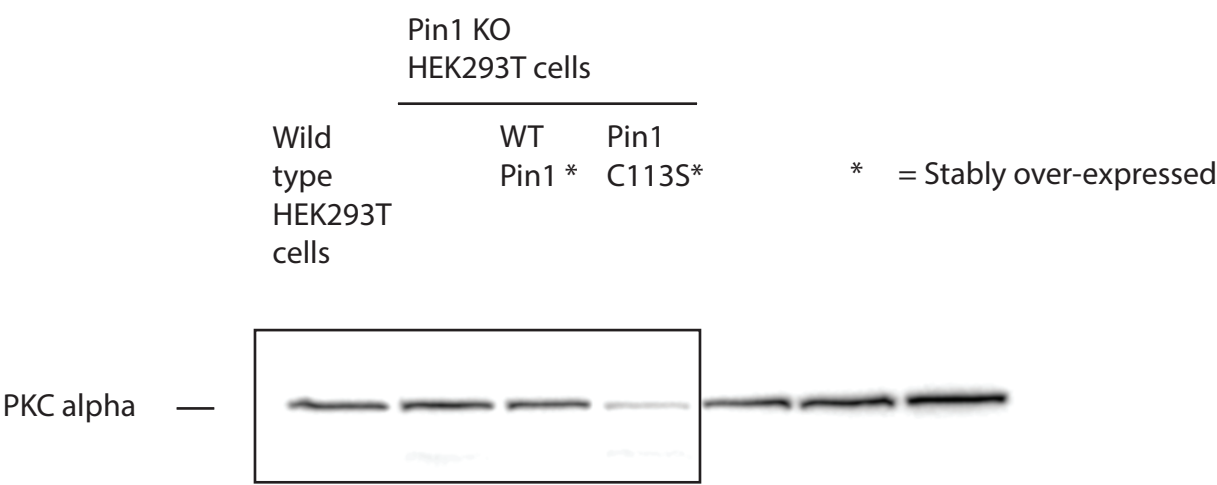

Supplement: Figure 7—source data 2. [file elife-92884-fig7-data2.zip › Figure 7-source data2/F7C Lower Panel/PKC alpha/F7C lower panel source data PKC labeled .pdf]

Figure 7 upper panel labeled PKC alpha source data

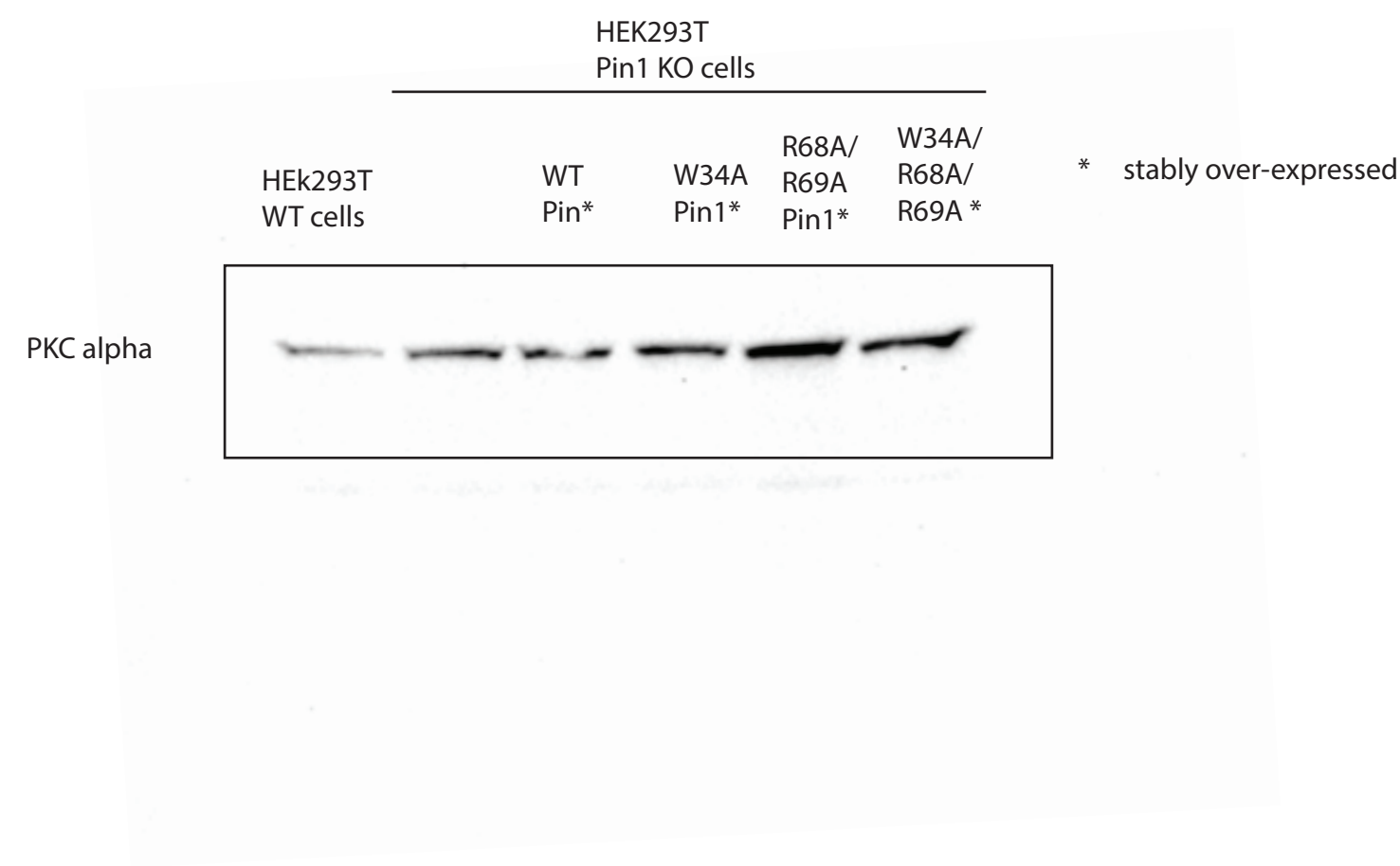

Supplement: Figure 7—source data 2. [file elife-92884-fig7-data2.zip › Figure 7-source data2/F7C Upper Panel/F7C Upper panel PKC/F7C Upper panel source data PKC alpha labeled.pdf]

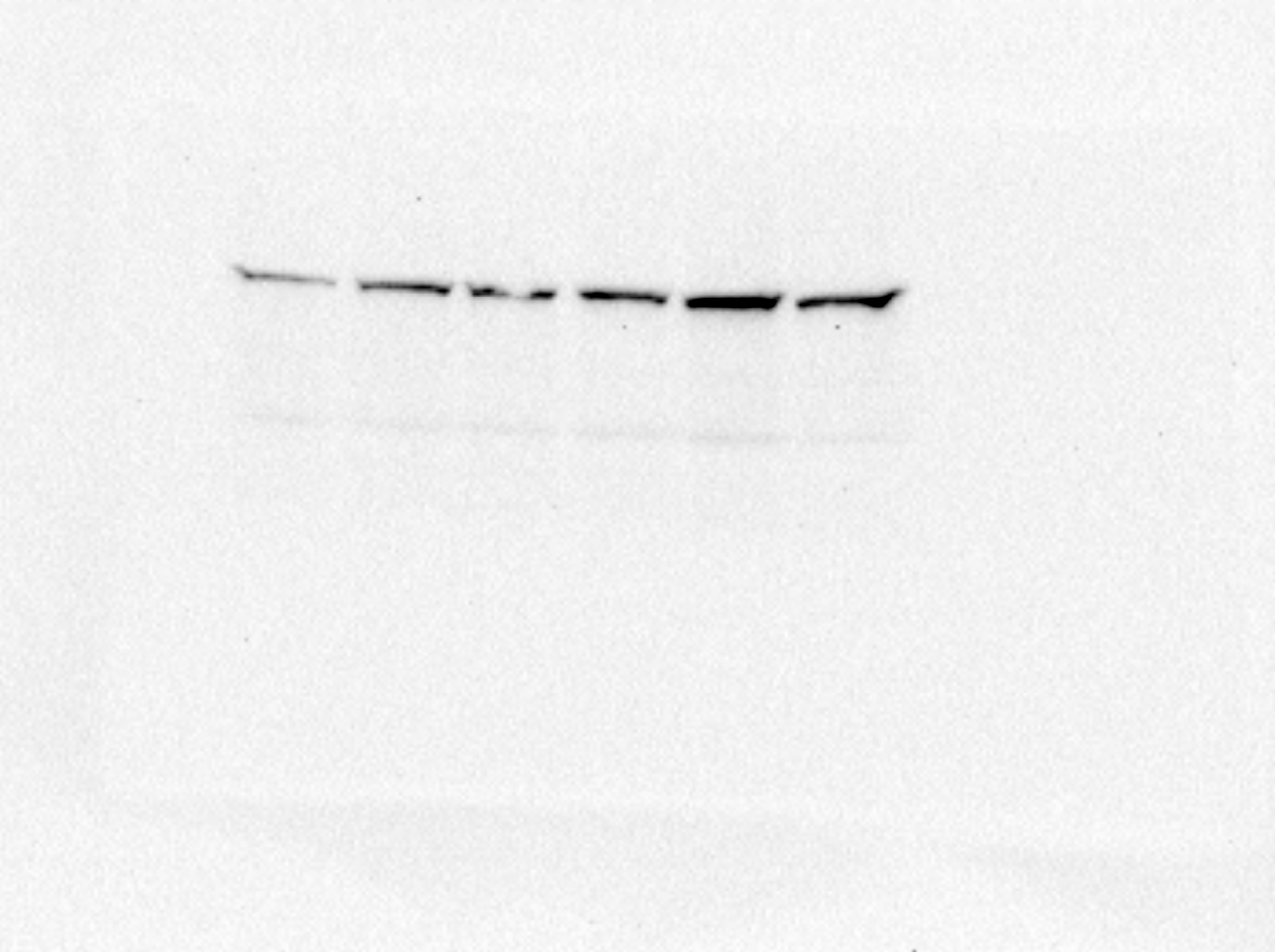

Supplement: Figure 7—source data 2. [file elife-92884-fig7-data2.zip › Figure 7-source data2/F7C Upper Panel/F7C Upper panel PKC/F7C Upper panel source data PKC.tif]

Figure 7C upper panel labeled actin source data

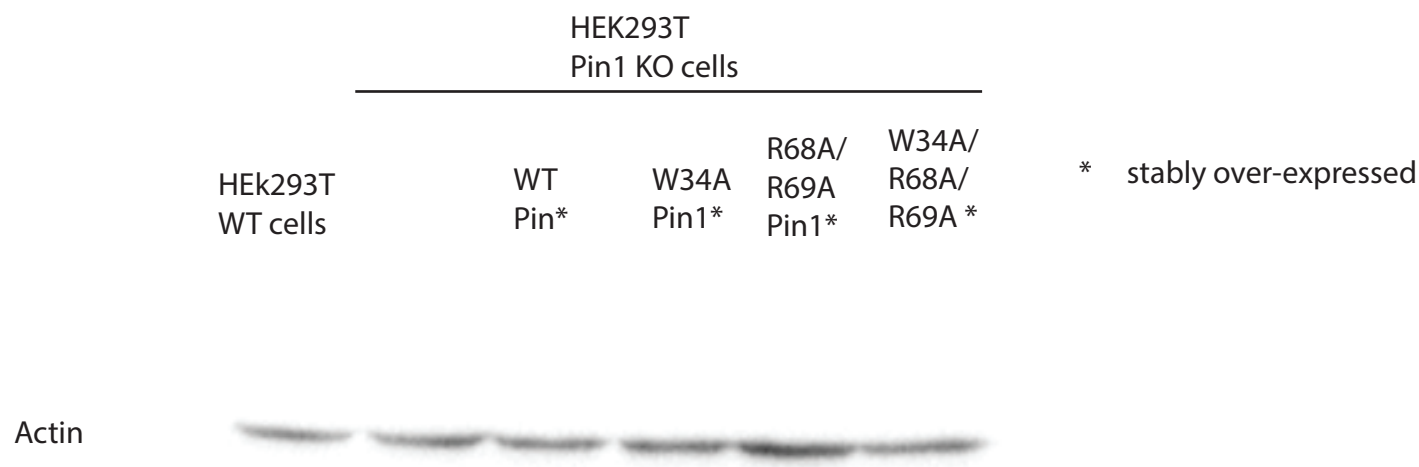

Supplement: Figure 7—source data 2. [file elife-92884-fig7-data2.zip › Figure 7-source data2/F7C Upper Panel/F7C Upper panel actin/F7C Upper panel source data actin labeled.pdf]

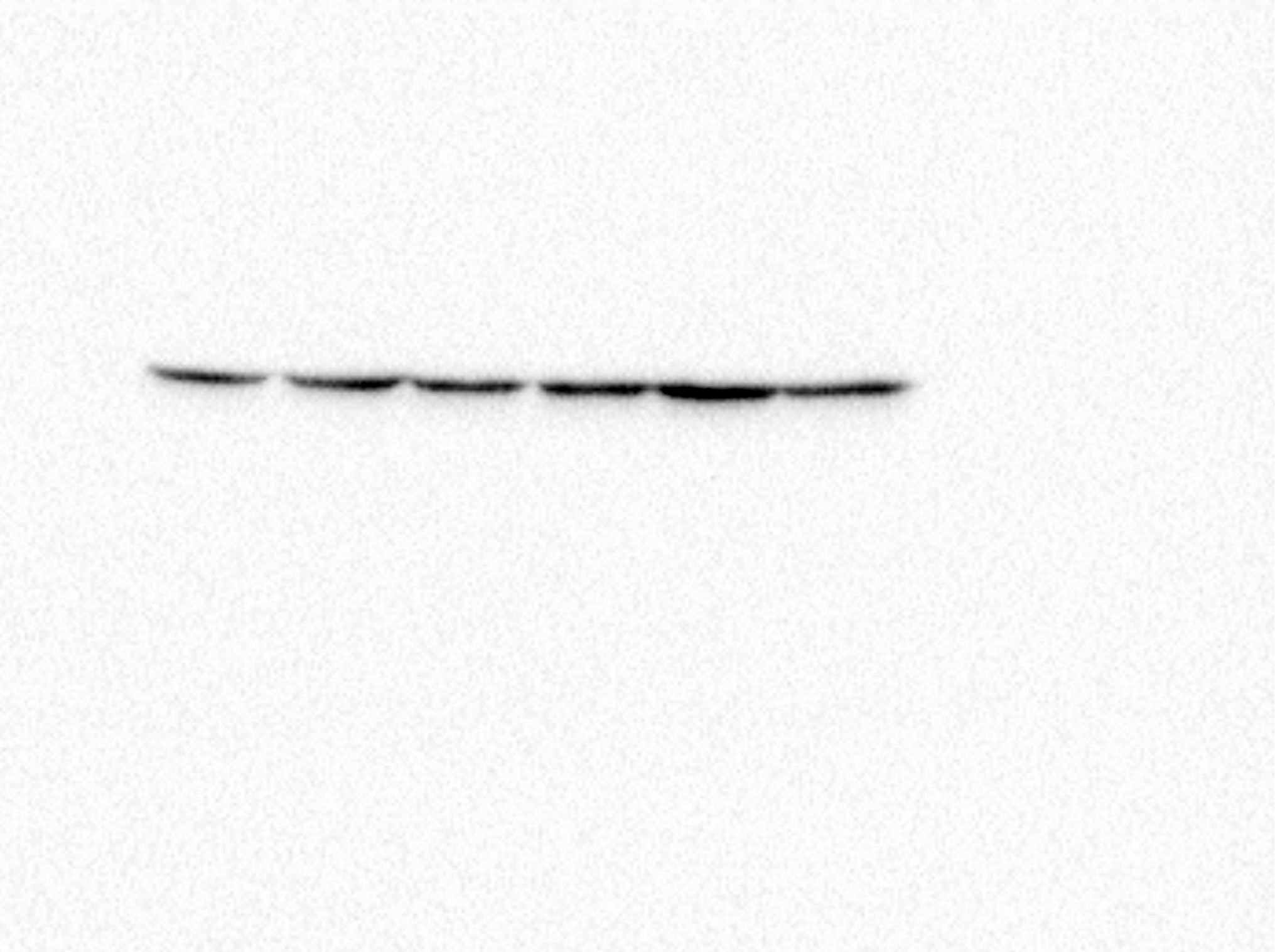

Supplement: Figure 7—source data 2. [file elife-92884-fig7-data2.zip › Figure 7-source data2/F7C Upper Panel/F7C Upper panel actin/actin raw data for pin1 manuscript.tif]

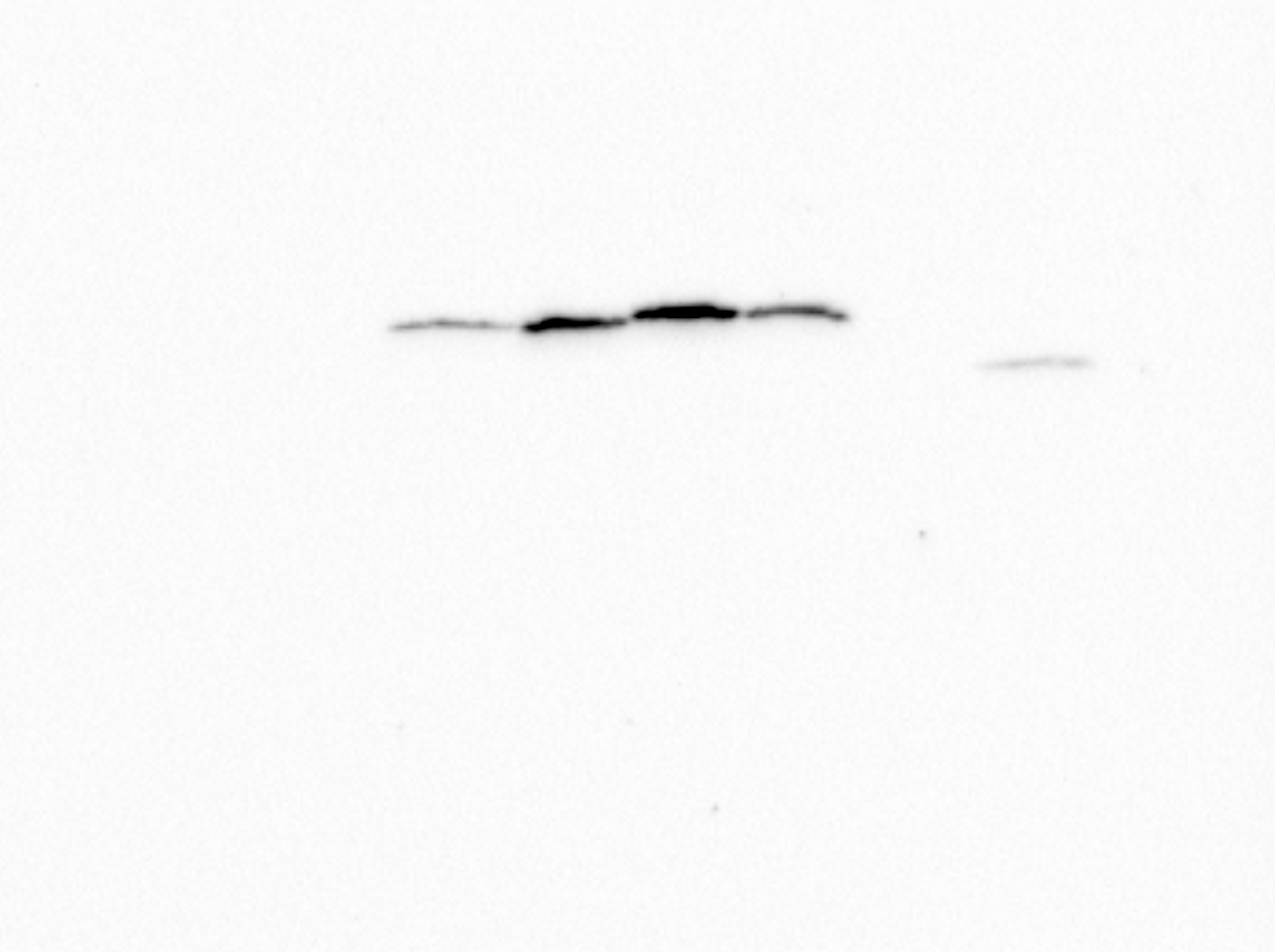

Supplement: Figure 7—source data 2. [file elife-92884-fig7-data2.zip › Figure 7-source data2/F7C Upper Panel/F7C Upper panel Pin1/F7C upper panel Pin1 source data.tif]

Figure 7C upper panel labeled Pin1 source data

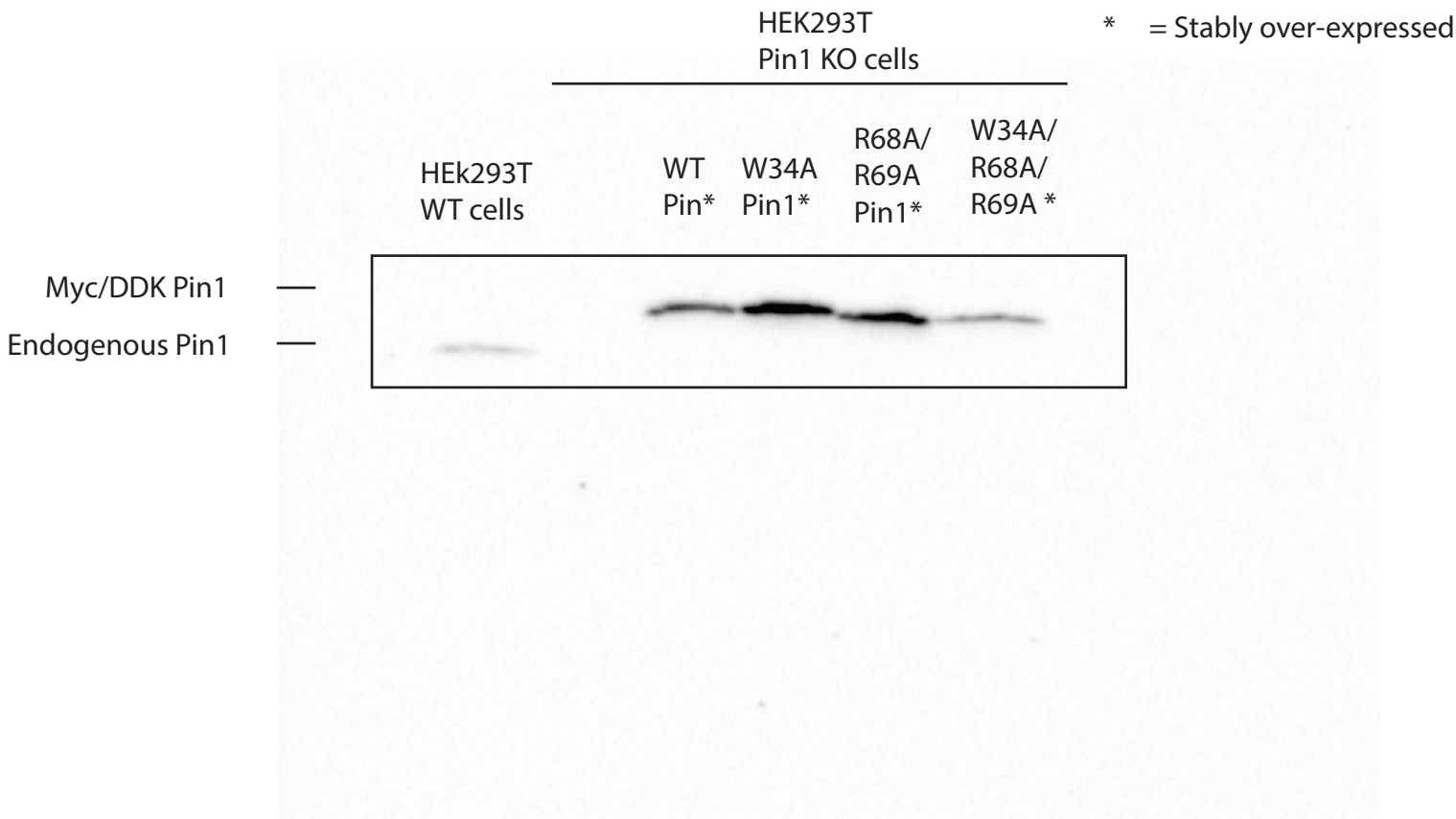

Supplement: Figure 7—source data 2. [file elife-92884-fig7-data2.zip › Figure 7-source data2/F7C Upper Panel/F7C Upper panel Pin1/F7C Upper panel source data Pin1 labeled.pdf]

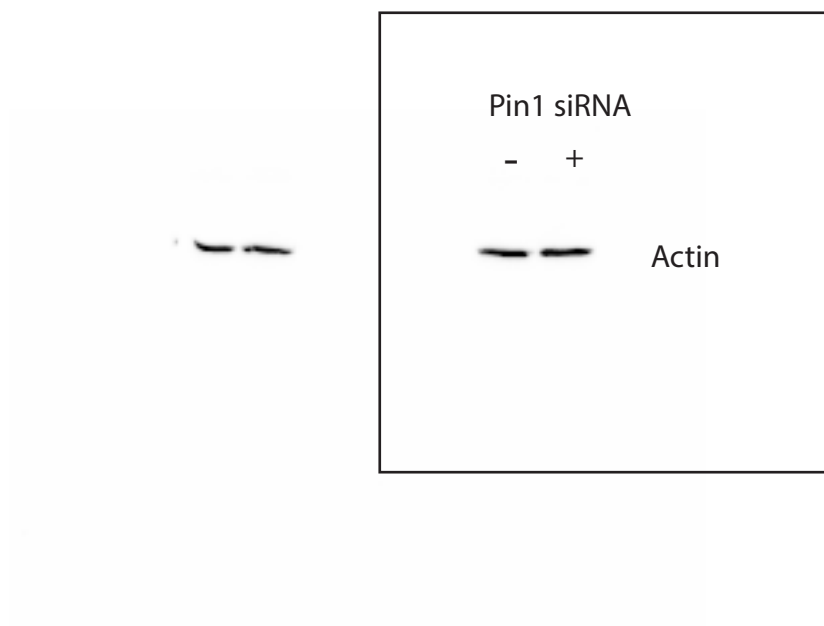

Supplement: Figure 7—figure supplement 1—source data 1. [file elife-92884-fig7-figsupp1-data1.zip › Figure 7-figure supplement 1_source data1/Figure 7-figure supplement 1A_Actin/Figure 7-figure supplement 1A_Actin source blot labeled.pdf]

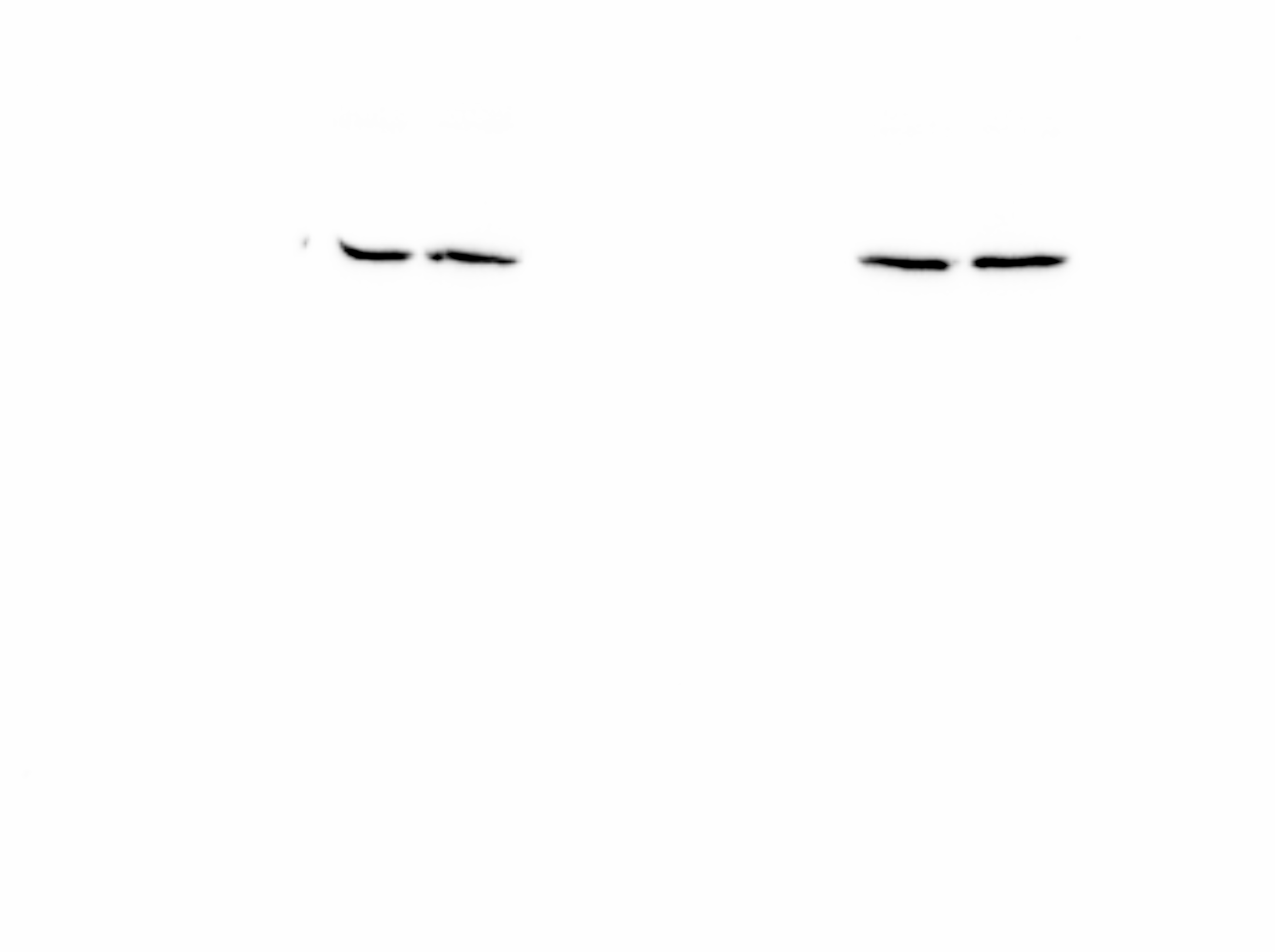

Supplement: Figure 7—figure supplement 1—source data 1. [file elife-92884-fig7-figsupp1-data1.zip › Figure 7-figure supplement 1_source data1/Figure 7-figure supplement 1A_Actin/Figure 7-figure supplement 1A_actin source unlabled.tif]

Pin1 siRNA

- +

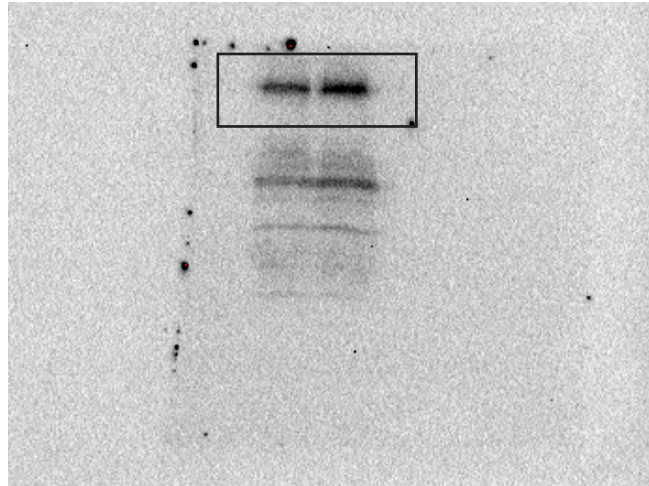

PKC $\alpha$

Supplement: Figure 7—figure supplement 1—source data 1. [file elife-92884-fig7-figsupp1-data1.zip › Figure 7-figure supplement 1_source data1/Figure 7-figure supplement 1_PKCa/Figure 7-figure supplement 1A_PKCa source blot labeled.pdf]

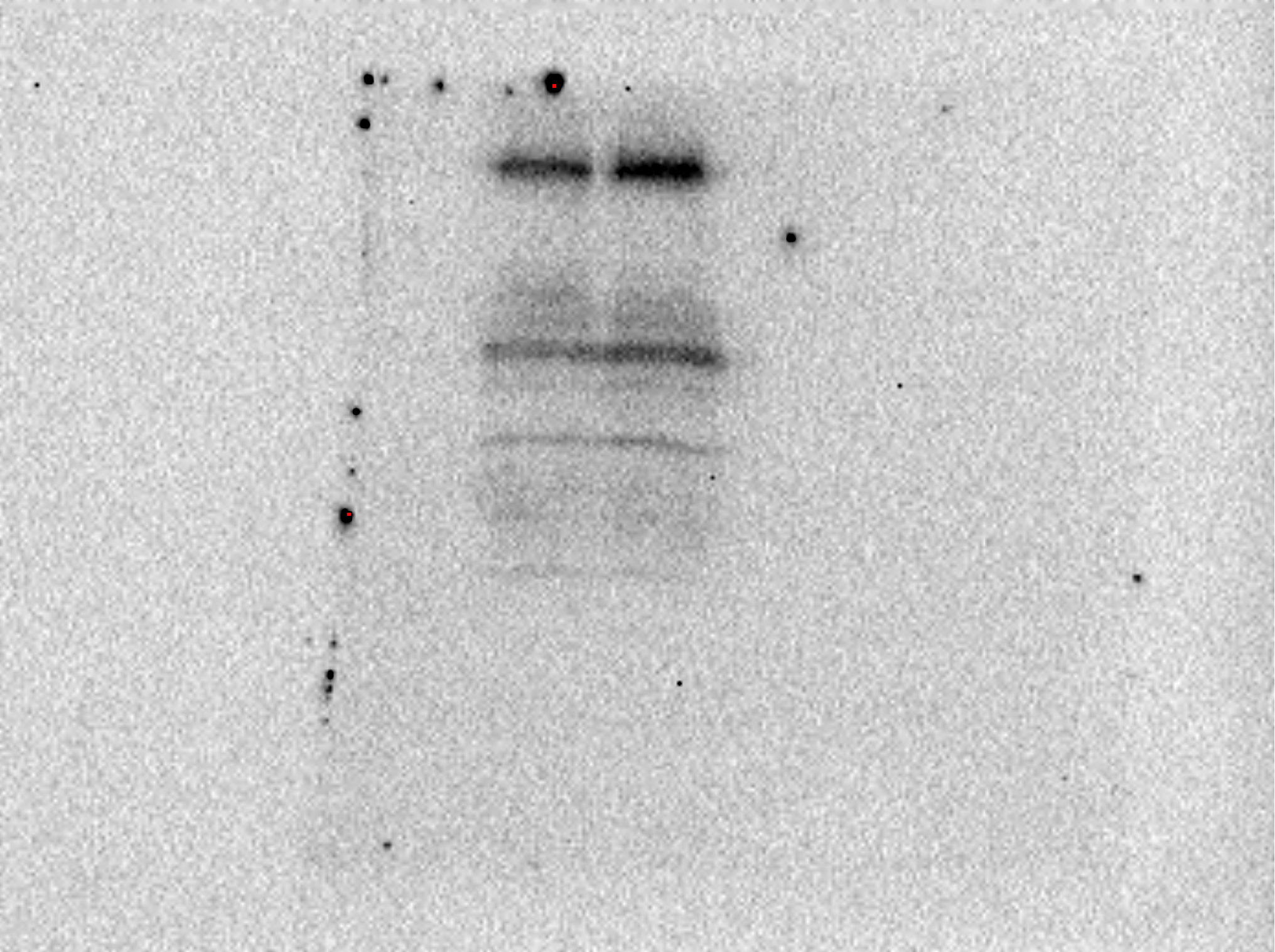

Supplement: Figure 7—figure supplement 1—source data 1. [file elife-92884-fig7-figsupp1-data1.zip › Figure 7-figure supplement 1_source data1/Figure 7-figure supplement 1_PKCa/Figure 7-figure supplement 1A_PKC alpha source unlabeled.tif]

Pin1 siRNA

- +

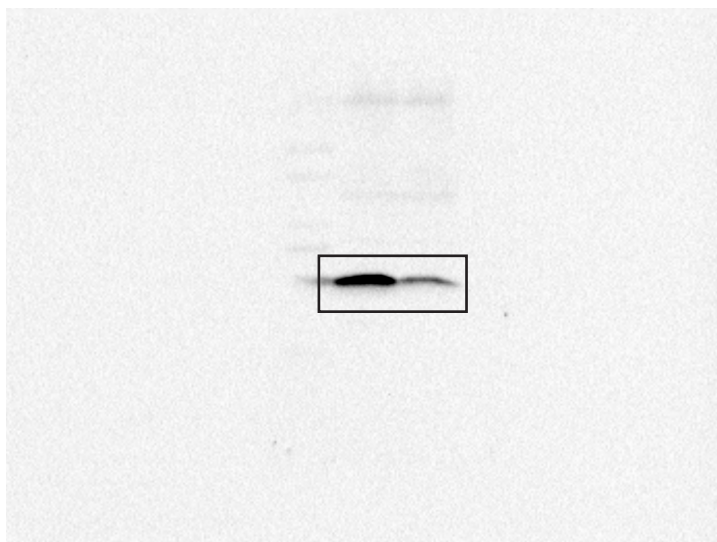

Pin1

Supplement: Figure 7—figure supplement 1—source data 1. [file elife-92884-fig7-figsupp1-data1.zip › Figure 7-figure supplement 1_source data1/Figure 7-figure supplement 1A_Pin1/Figure 7-figure supplement 1A_Pin1 source blot labeled.pdf]

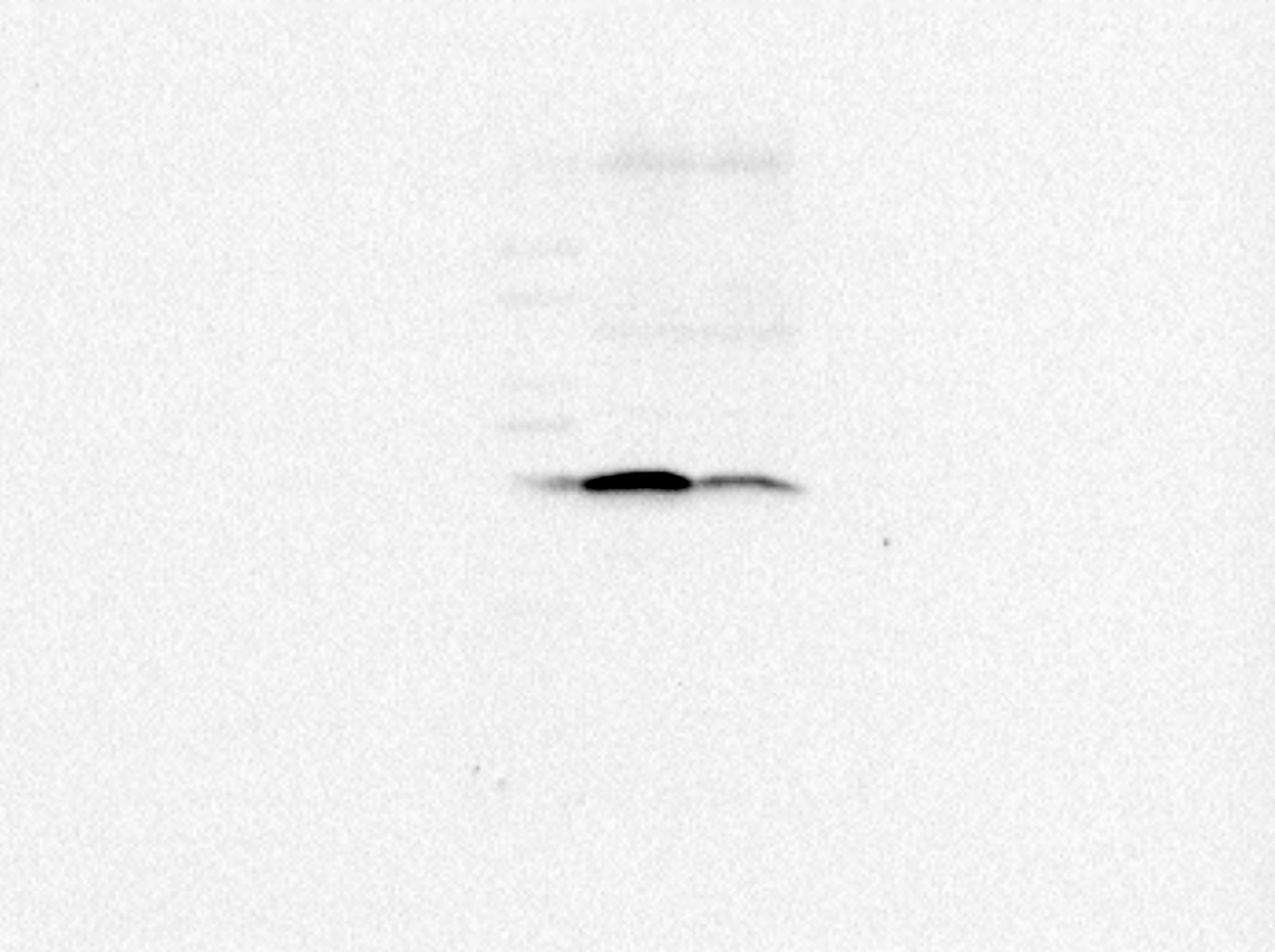

Supplement: Figure 7—figure supplement 1—source data 1. [file elife-92884-fig7-figsupp1-data1.zip › Figure 7-figure supplement 1_source data1/Figure 7-figure supplement 1A_Pin1/Figure 7-figure supplement 1Apin1 source unlabeled 2.tif]
