# Supplementary material for: A novel bivalent interaction mode underlies a non-catalytic mechanism for Pin1-mediated protein kinase C regulation
Source: eLife. 2024 Apr 30;13:e92884. doi: 10.7554/eLife.92884 (PMC11060717; doi:10.7554/eLife.92884)
Supplement: Supplementary file 1. — All peptides have acetylated N-termini and amidated C-termini. TM and HM stand for the turn and hydrophobic motifs, respectively. The phosphorylated Thr of the TM and phosphorylated Ser of the HM are shown in red. Peptides having ‘V5’ in their name contain both TM and HM. Peptides starting with ‘p’ indicate that the peptide is phosphorylated at either one or both motifs, the latter only for the ‘V5’ peptides. [file elife-92884-supp1.docx]

| **Source** | **Peptide name** | **Sequence** | **pI** | **Solu-bility**  **(water)** | **Vendor name** |
| --- | --- | --- | --- | --- | --- |
| **PKCα** | 1. V5α | ^632^RGQPVLT^638^PPDQLVIANIDQSDFEGFS^657^YVN^660^ | 3.3 | Poor | Eton-Bioscience |
|  | 2. V5α-pTM-HM | ^632^RGQPVLpT^638^PPDQLVIANIDQSDFEGFS^657^YVN^660^ | 3.3 | Poor | Eton-Bioscience |
|  | 3. V5α-TM-pHM | ^632^RGQPVLT^638^PPDQLVIANIDQSDFEGFpS^657^YVN^660^ | 3.3 | Poor | Eton-Bioscience |
|  | 4. pV5α | ^632^RGQPVLpT^638^PPDQLVIANIDQSDFEGFpS^657^YVN^660^ | 3.3 | Poor | Eton-Bioscience |
|  | 5. pTMα | ^632^RGQPVLpT^638^PPDQ^642^ | 7.9 | Good | Eton-Bioscience |
|  | 6. pHMα | ^646^ANIDQSDFEGFpS^657^YVN^660^ | 0 | Poor | Eton-Bioscience |
| **PKCβII** | 7. V5βII | ^640^LT^641^PPDQEVIRNIDQSEFEGFS^660^F^661^ | 3.3 | Good | Eton-Bioscience |
|  | 8. pV5βII | ^640^LpT^641^PPDQEVIRNIDQSEFEGFpS^660^F^661^ | 3.3 | Good | Eton-Bioscience  Thermo-Fisher Scientific |
|  | 9. pTMβII | ^640^LpT^641^PPDQEVIR^649^ | 3.9 | Good | Eton-Bioscience |
|  | 10. pHMβII | ^650^NIDQSEFEGFpS^660^F^661^ | 0 | Good | Eton-Bioscience |
|  | 11. V5βII-TM-pHM | ^640^LT^641^PPDQEVIRNIDQSEFEGFpS^660^F^661^ | 3.3 | Good | Eton-Bioscience |
|  | 12. V5βII-pTM-HM | ^640^LpT^641^PPDQEVIRNIDQSEFEGFS^660^F^661^ | 3.3 | Good | Thermo-Fisher Scientific |
|  | 13. Ext-pV5bII | ^639^VLpT^641^PPDQEVIRNIDQSEFEGFpS^660^FVN^663^ | 3.3 | Good | Thermo-Fisher Scientific |
|  | 14. Labeled-pTMβII | ^640^LpT^641^PPDQEVIR^649^ (^13^C,^15^N-labeled) | 3.9 | Good | Sigma-Aldrich |
|  | 15. Labeled-pHMβII | ^650^NIDQSEFEGFpS^660^F^661^ (^13^C,^15^N-labeled) | 0 | Good | Sigma-Aldrich |
| **α, βII** | 16. SP-1 | LpTPPD | 0 | Good | Eton-Bioscience |
| **βI** | 17. SP-2 | LpTPTD | 0 | Good | Eton-Bioscience |
| **α, variant** | 18. pTMα P640A | ^632^RGQPVLpT^638^PADQ^642^ | 7.9 | Good | Eton-Bioscience |
