# Supplementary material for: A novel bivalent interaction mode underlies a non-catalytic mechanism for Pin1-mediated protein kinase C regulation
Source: eLife. 2024 Apr 30;13:e92884. doi: 10.7554/eLife.92884 (PMC11060717; doi:10.7554/eLife.92884)
Supplement: Supplementary file 2. [file elife-92884-supp2.docx]

| **Binding Experiment** | **Protein** | **V5 region,**  **Max conc. (µM)** | **Residues selected for analysis** | **K_d_ (µM)**  **Chem. shift** | **K_d_ (µM)**  **Lineshape**  **analysis** |
| --- | --- | --- | --- | --- | --- |
| 1 | Pin1 | pTMβII, 1000 | K13, R14, M15, S16, G20, R21, V22, Y23, Y24, F25, N26, I28, N30, A31, S32, Q33, W34, E35, R36 | 9.1 ± 0.4 | 9.6 ± 0.1 |
| 2 | WW | pTMβII, 1000 | W34, S16, E35, Q33, G20, K13, H27, M15, N26, R14, S32, Y24, V22, A31, F25, Y23, T29, W34ε, R21, N30, E12, I28 | 14.3 ± 0.2 | 16.0 ± 0.1 |
| 3 | Pin1 | pTMα, 1800 | R36, Q33, M15, K13, R21, N26, I28, F25, E35, G20, Y24, V22, E12, R14 | 35.4 ± 0.6 | 32.7 ± 0.1 |
| 4 | WW | pTMα, 1800 | S32, S16, A31, E35, N26, N30, Q33, M15, G20, R14, K13, Y24, Y23, W34, V22 | 25.2 ± 0.2 | 24.0 ± 0.1 |
| 5 | WW | pHMα, 1800 | Q33, S32, W34ε, W34, E35, F25, V22, S16, G20, Y24, A31, N30, Y23, T29 | 1006 ± 9 |  |
| 6 | PPIase | pHMα, 1878 | A116, A124, D136, D153, E135, F125, F134, G123, H59, K117, K132, M130, Q129, Q131, S114, S115, S126, W73, R68, R69 | 835 ± 20 |  |
| 7** | Pin1 | pHMα, 1800 | δH and δN: S18, G20, V22, Y23, Y24, F25, T29, N30, S32, Q33, W34, W34 ε, E35, S58, S72, S114, S115, A116, K117, G128, Q129, Q131, K132, D136, T152, D153;  δH only: Q66, R68, R69, G120, F125, M130, S154;  δN only: H59, Q66, R68, R69, W73, W73ε, K82, D121, A124, F125, S126, F134, E135, A31 | **WW:** 725 ± 9  **PPIase:** 701 ± 7 |  |
| 8 | WW | pHMβII, 2550 | W34, S16, E35, Q33, G20, S32, Y24, V22, A31, F25, Y23, T29, N30, W34 ε | 782 ± 9 | 809 ± 4 |
| 9 | PPIase | pHMβII, 1125 | S65, I156, L61, V55, A124, K117, V150, L141, L60, R127, S138, T152, S72, E135, G155, Q129, H59, S126, G128, F125, D136, S154, K132, F134, R69, S115 | 133 ± 1 | 127 ± 1 |
| 10** | Pin1 | pHMβII, 1350 | δH and δN: G20, V22, Y24, F25, A31, S32, Q33, W34, E35, R54, V55, H59, Q66, R68, R69, S72, K82, S114, S115, K117, A124, F125, S126, R127, Q129, K132, F134, E135, D136, S138, F139, L141, V150, T152, I156;  δH only: T29, R36, L61, S65, W73, L122, G128;  δN only: N30, G123, G155 | **WW:** 257 ± 9  **PPIase:** 75.0 ± 1.1 |  |
| 11 | Pin1 | V5βII-pTM-HM, 460 | W34, S18, E35, S16, Q33, G20, R14, K13, M15, N26, V22, Y24, N30, F25, A31, I28, S32, E12, Y23, T29, R21, R36, S115, F134, F139, L141, E51, R54, R142, A140, A53, V55, I158, S114, S126, Q129, E135, Q131, M130, F125, G128, R69, T152, K117 | 21.0 ± 0.5 | 20.1 ± 0.1 |
| 12** | Pin1 | V5α-TM-pHM,  1320 | δH and δN: V22, Y24, A31, S32, W34, E35, R69, S72, S114, S115, A116, K117, F125, G128, M130, Q131, L141, T152, D153;  δH only: G20, F25, T29, H59, W73, K82, S126, F134, E135;  δN only: Q33, S55, R68, G123, Q129, S154 | **WW:** 688 ± 21  **PPIase:** 757 ± 16 |  |
| 13** | Pin1 | V5βII-TM-pHM,  1640 | δH and δN: G20, V22, Y24, F25, A31, S32, Q33, W34, V55, H59, Q66, S72, K82, S114, S115, K117, A124, F125, S126, R127, Q129, K132, F134, E135, D136, S138, F139, L141, V150, T152, I156;  δH only: T29, E35, R36, R54, L61, S65, R68, R69, W73, L122, G128;  δN only: N30, R54, R68, R69, L122, G123, G155 | **WW:** 213 ± 7  **PPIase:** 93.6 ± 1.5 |  |
| 14 | Pin1 | pV5α, 1530 | A31, F25, G20, K13, M15, N26, N30, Q33, R14, R21, S32, T29, V22, Y23, Y24, A116, E135, E51, F125, F134, F139, G123, G128, K117, K82, L141, M130, Q129, Q131, R68, R69, S114, S115, S126, S71, S72, T152, W73ε, W73 | 16.0 ± 0.6 | 12.0 ± 0.2 |
| 15 | Pin1 | pV5βII, 400 | M15, V22, F25, N26, A31, S32, R54, V55, S72, S115, K117, G128, Q129, D136, F139, L141, S147, V150, T152, S154, G155, R69, S115, K132, F125, S126 | N/D | 1.5 ± 0.1 |
| 16 | Pin1 | V5βII, 1650 | K13, S16, G20, V22, T29, A31, S32, Q33, W34, E35, R68, W73, K112, S114, S115, A116, D121, G123, A124, F125, S126, G128, Q129, M130, Q131, E135, D136, L141, T152, G155 | 964 ± 34 |  |
| 17 | Pin1 | V5α, 500 |  | N/A |  |
| 18 | Pin1, pTMβII-complexed (98% saturated) | pHMβII, 500 | S65, V55, A124, K117, V150, L141, L60, S138, T152, S72, E135, G155, Q129, H59, S126, G128, F125, D136, S154, K132, F134, R69, S115, G123, A107, Q131, S114 | 117 ± 2 |  |
| 19 | Pin1, C113S variant | pV5βII, 350 | E12, R14, M15, F25, N26, T29, A31, R54, H64, S72, D112, A116, F125, S126, G128, Q129, M130, F134, A137, F139, L141, S147 | N/D | 3.4 ± 0.1 |

**2-site binding model

N/D: Not determined; affinity is too high for the chemical-shift based analysis.

N/A: Impossible to determine the K_d_ due to very low affinity.
