# Supplementary material for: A novel bivalent interaction mode underlies a non-catalytic mechanism for Pin1-mediated protein kinase C regulation
Source: eLife. 2024 Apr 30;13:e92884. doi: 10.7554/eLife.92884 (PMC11060717; doi:10.7554/eLife.92884)
Supplement: Supplementary file 3. — Sample 2* was prepared in the buffer containing 100% D2O. [file elife-92884-supp3.docx]

| **ID** | **Complex** | **pV5βII conc. (mM)** | **Pin1 conc. (mM)** | **Bound pV5βII,%** | **Bound Pin1, %** | **Experiments** |
| --- | --- | --- | --- | --- | --- | --- |
| 1 | [U-^13^C, ^15^N] Pin1 + pV5βII | 1.05 | 0.9 | 98 | 84 | --2D experiments: [^15^N, ^1^H] HSQC, ct [^13^C, ^1^H] HSQC, (HB)CB(CGCDCE)HE, (HB)CB(CGCD)HD  --3D experiments for backbone assignment: HNCACB, CBCA(CO)NH, HNCO, HN(CA)CO  --3D experiments for side-chain assignments: C(CO)NH, H(CCO)NH, HNHA, HNHB  --NOESY experiments: 3D ^15^N-edited NOESY-HSQC, 3D [F1] ^13^C,^15^N-filtered NOESY-^15^N-HSQC |
| 2* | [U-^13^C, ^15^N] Pin1 + pV5βII | 1.05 | 0.9 | 98 | 84 | --^1^H/^2^D exchange  --2D ct [^13^C, ^1^H] HSQC, 2D ct ^1^H-^13^C aromatic HSQC  --3D experiments for sidechain assignments: HCCH-COSY, HCCH-TOCSY  --NOESY experiments: 3D ^13^C-edited NOESY-HSQC, 3D C^aro^-edited NOESY-HSQC, 3D [F1] ^13^C,^15^N-filtered NOESY-^13^C-HSQC |
| 3 | [U-^13^C, ^15^N] Pin1 + pV5βII | 1.0 | 1.3 | 76 | 99 | --2D [F1] ^13^C,^15^N-filtered NOESY  --2D [F2] ^13^C,^15^N-filtered NOESY  --2D [F1, F2] ^13^C,^15^N-filtered NOESY  --2D [F1, F2] ^13^C,^15^N-filtered TOCSY |
| 4 | [U-^13^C, ^15^N] PPIase + pHMβII | 0.8 | 5.5 | 97 | 14 | --3D [F1] ^13^C, ^15^N-filtered NOESY-^15^N-HSQC  --3D [F1] ^13^C, ^15^N-filtered NOESY-^13^C-HSQC |
| 5 | [U-^13^C, ^15^N] PPIase + pHMβII | 0.5 | 1.8 | 25 | 91 | --2D [F1] ^13^C,^15^N-filtered NOESY  --2D [F2] ^13^C,^15^N-filtered NOESY  --2D [F1, F2] ^13^C,^15^N-filtered NOESY  --2D [F1, F2] ^13^C,^15^N-filtered TOCSY |
| 6 | PPIase + [U-^13^C,^15^N Phe] pHMβII  *peptide ID #15 (Supplementary File 1)* | 0.5 | 2.5 | 19 | 93 | --3D [F1] ^13^C,^15^N-filtered NOESY-^15^N-HSQC  --3D [F1] ^13^C,^15^N-filtered NOESY-^13^C^aro^-HSQC  --3D [F1] ^13^C,^15^N-filtered NOESY-^13^C^ali^-HSQC |
| 7 | Pin1 + [U-^13^C,^15^N Pro, Ile, Val] pTMβII  *peptide ID #14 (Supplementary File 1)* | 1.0 | 1.5 | 66 | 98 | --3D [F1] ^13^C,^15^N-filtered NOESY-^15^N-HSQC  --3D [F1] ^13^C,^15^N-filtered NOESY-^13^C-HSQC |
| 8 | [U-^13^C, ^15^N] Pin1 + Ext-pV5βII  *peptide ID #13 (Supplementary File 1)* | 1.3 | 1.0 | >95% |  | --3D [F1] ^13^C,^15^N-filtered NOESY-^15^N-HSQC  --3D [F1] ^13^C,^15^N-filtered NOESY-^13^C-HSQC |
| 9 | [U-^13^C, ^15^N] Pin1 + Ext-pV5βII  *peptide ID #13 (Supplementary File 1)* | 0.8 | 1.3 |  | >95% | --2D [F1] ^13^C,^15^N-filtered NOESY  --2D [F2] ^13^C,^15^N-filtered NOESY  --2D [F1, F2] ^13^C,^15^N-filtered NOESY  --2D [F1, F2] ^13^C,^15^N-filtered TOCSY |
