# Supplementary material for: A novel bivalent interaction mode underlies a non-catalytic mechanism for Pin1-mediated protein kinase C regulation
Source: eLife. 2024 Apr 30;13:e92884. doi: 10.7554/eLife.92884 (PMC11060717; doi:10.7554/eLife.92884)
Supplement: Supplementary file 4. [file elife-92884-supp4.docx]

| **No. of NOE restraints for Pin1** | |
| --- | --- |
| No. of NOE-based distance restraints for Pin1 |  |
| Total | 2780 |
| Intra-residue (\|i – j\| = 0) | 1071 |
| Sequential (\|i – j\| = 1) | 624 |
| Medium-range (1< \|i – j\| ≤ 4) | 339 |
| Long-range (\|i – j\| ≥ 5) | 662 |
| NOE restraints per residue | ~17 |
| No. of hydrogen bond restraints | 84 |
| No. of dihedral angle restraints | 244 |
| Total no. of restraints | 3024 |
| Total no. of restraints per residue | ~18.6 |
| **No. of NOE restraints for pV5βII** | |
| No. of NOE-based distance restraints for pV5βII |  |
| Total | 241 |
| Intra-residue (\|i – j\| = 0) | 147 |
| Sequential (\|i – j\| = 1) | 67 |
| Medium-range (1< \|i – j\| ≤ 4) | 24 |
| Long-range (\|i – j\| ≥ 5) | 3 |
| NOE restraints per residue | ~11.0 |
| No. of dihedral angle restraints | 0 |
| Total no. of restraints | 241 |
| Total no. of restraints per residue | 11 |
| **Total No. of intermolecular NOEs = 75** | |
| Total no. of structures calculated | 500 |
| Total no. of structures refined | 50 |
| No. of structures used | 20 |
| No. of restraint violations |  |
| Dihedral angle of > 5° |  |
| Distance of > 0.2 Å |  |
| Van der Waals | 149 |
| Ramachandaran plot (Procheck) |  |
| Most favoured regions (%) | 89.3 |
| Additionally allowed regions (%) | 10.7 |
| Generously allowed regions (%) | 0.0 |
| Total allowed regions (%) | 100.0 |
| Disallowed regions (%) | 0.0 |
| Ramachandaran plot (Molprobity) |  |
| Most favoured regions (%) | 96.2 |
| Allowed regions (%) | 3.8 |
| Total allowed regions (%) | 100.0 |
| Disallowed regions (%) | 0.0 |
| RMSD from mean structure coordinates (Å) |  |
| Backbone | 0.9 |
| Average heavy atom | 1.2 |
